# Evaluation of deletion of nuclease genes cluster in *L. major*

**DOI:** 10.1186/1753-6561-5-S1-P39

**Published:** 2011-01-10

**Authors:** Noushin Davoudi, Maryam Hejazi, Zahra Khodayari, Robert W McMaster

**Affiliations:** 1 Research Center of Biotechnology, Department of Biotechnology, Pasteur Institute of Iran, Tehran, 13169, Iran; 2 Department of Medical Genetics, University of British Columbia, Vancouver Hospital, Vancouver, British Columbia, Canada V6H 3Z6

## 

The nuclease is a surface enzyme unique to trypanosomatid parasites. These organisms lack the pathway for de novo purine biosynthesis and thus are entirely dependent upon their hosts to supply this nutrient for their survival, growth, and multiplication. There is a cluster on chromosome 30 which carries 2 copy of nuclease genes and 5 identical nuclease like proteins in *L. major* which are in *cis* form with 700-800 bp intergenic regions which have more than 80% homology. These data shows that this enzyme might play an important role in facilitating the survival, growth, and development of this important human pathogen. In previous studies, have been shown that *L. major* 3′NT/NU which is expressed specifically in promastigotes is not the key molecules involved in host purine salvaging pathway and thus in better understanding parasite strategies adopted to survive in sandflies. Therefore, only deletion of nuclease genes followed by sandfly infections experiments will allow determining its precise role in purine salvaging and sandfly infection and specificity. We have developed nuclease cluster heterozygote and homozygote knockout mutants, with homologous recombination technique, to evaluate deletion effects on survival of parasite and infection.

